# Supplementary material for: Occupational socialization in pre-service physical education teachers
Source: PLoS One. 2022 Jul 21;17(7):e0271772. doi: 10.1371/journal.pone.0271772 (PMC9302786; doi:10.1371/journal.pone.0271772)
Supplement: Attachment — Submitted filename: Response to Reviewers.doc [file pone.0271772.s001.doc]

Dear editor and reviewers,

      I appropriate your approval and comments on my paper. I will respond to each comment/question following the sequence of the email I received.

To the editor/Journal requirements:

1. I have modified my paper based on PLOS ONE’s sample.
2. I added some details about the ethic statement in the manuscript. Please see page 5, lines 9-10.
3. I have updated the raw data onto Figshare. Please check them from this website: <https://doi.org/10.6084/m9.figshare.20170820.v1>I have reviewed the references list to avoid any inappropriate citations.

To Dr. Ertan Tufekcioglu/reviewer #1:

1. I have modified the sentences about the university’s approval of this study. Please see page 9, lines 4-9.
2. Under the Trustworthiness, “PCK” was a typo that I have modified. Please see page 11, line 3.

To Dr. Bijen Filiz/reviewer #2:

1. I have modified PETE to physical education teacher education in the abstract. Please see page 1, lines 16-17.
2. By considering your comment, I have removed the mix-methods part but explain the quantitative and qualitative research methods separately. Please see page 1, line 11-12; page 4, line 19-20; page 5, line 1-3; page 20, line 13.
3. I did not ask the participants to support their ages but only their grades. Age was not a critical impactor in my study. The participants in different grades would have different academic and teaching experiences which were considered as a critical factor to influence their OS development.
4. I have added the information about the validity of the original and modified survey. Please see page 5, line 20; page 6, line 1. I also added the reliability test of the modified survey. Please see page 6, line 18-20; page 7, line 1-7.
5. I added some details about the interview guideline. Please see page 8, lines 4-6.
6. I added some details about the qualitative data analysis process. Please see page 10, line 10-17; page 11, line 7-8.

I also made some modifications to the typos and other things I think should be changed. Please see the Revised Manuscript with Track Changes file. Please let me know if there is anything else I need to do.

Again, thank you so much for your help!

Sincerely,

Dong
